# A greedy stacking algorithm for model ensembling and domain weighting

**DOI:** 10.1186/s13104-020-4931-7

**Published:** 2020-02-12

**Authors:** Christoph F. Kurz, Werner Maier, Christian Rink

**Affiliations:** 1 Institute of Health Economics and Health Care Management, Helmholtz Zentrum München, Ingolstädter Landstraße 1, Neuherberg, Germany; 2 MAN Truck & Bus AG Munich, Elisabeth-Selbert-Strasse 1, 80939 München, Germany

**Keywords:** Model ensembling, Greedy algorithm, Optimization, Machine learning

## Abstract

**Objective:**

Because it is impossible to know which statistical learning algorithm performs best on a prediction task, it is common to use stacking methods to ensemble individual learners into a more powerful single learner. Stacking algorithms are usually based on linear models, which may run into problems, especially when predictions are highly correlated. In this study, we develop a greedy algorithm for model stacking that overcomes this issue while still being very fast and easy to interpret. We evaluate our greedy algorithm on 7 different data sets from various biomedical disciplines and compare it to linear stacking, genetic algorithm stacking and a brute force approach in different prediction settings. We further apply this algorithm on a task to optimize the weighting of the single domains (e.g., income, education) that build the German Index of Multiple Deprivation (GIMD) to be highly correlated with mortality.

**Results:**

The greedy stacking algorithm provides good ensemble weights and outperforms the linear stacker in many tasks. Still, the brute force approach is slightly superior, but is computationally expensive. The greedy weighting algorithm has a variety of possible applications and is fast and efficient. A python implementation is provided.

## Introduction

It is generally impossible to know a priori which learning algorithm (e.g., Random Forest, linear regression) performs best for a particular prediction task. For this reason, researchers have proposed combining different learners to build a powerful single learner. These methods are called *stacking*, *stacked regression*, or *super learning* in the literature [1–3].

The principle of stacking can be explained like this: given *d* different learning algorithms, evaluate each of them on the predictor matrix *X*, given outcome vector *y* in a *k*-fold cross-validation. Save the out-of-fold predictions and combine them to a new data matrix *Z*. *Z* now has *d* columns and the same number of rows as *X*. Then, estimate a weighted scheme for each column of *Z* to combine to a final prediction. A more detailed description of the stacking principle, including a graphical overview, can be found in [4–6].

This paper is motivated by the discussion on how the weighting of the single learners should be assessed. Van der Laan et al. suggest using a constrained linear regression model, so that the coefficients $\beta$ in the linear model are positive and sum to 1 [3]. They discuss that this has potential problems if the predictions in *Z* are collinear, yielding problems in both the interpretability and the numerical instability of linear models. In this paper, we develop a greedy algorithm to produce weights to optimally combine predictions of the single learners that overcomes collinearity issues and is easily interpretable. Additionally, because of its greedy nature, the stacked predictions will always be at least as good as the best single learner in the ensemble [7, 8]. We evaluate our greedy stacking algorithm on prediction tasks using different data sets from a variety of biomedical disciplines. In a second example, we show that this algorithm can be used in further applications. We look at optimizing the weights of the single domains of the German Index of Multiple Deprivation (GIMD) [9, 10] to be highly correlated with mortality. There is good evidence that mortality is associated with regional deprivation in European countries [11, 12], but the weighting of the domains of deprivation (e.g., income, education) that build the GIMD is based on expert knowledge.

## Main text

### Methods

In the following, we provide a description of the proposed greedy weighting algorithm in pseudocode. Two inputs have to be provided: a matrix *Z*, where each column represents a single feature for weighting, and a vector *y* for designated values to be weighted for. The columns in *Z* can be predictions from different learners, or for example, the different domains (i.e. vectors that indicate area-level deprivation in various socio-economic spheres) that build the GIMD. Furthermore, a function *metric* is needed as an evaluation metric (e.g., AUROC, correlation). The weights will be optimized in order to maximize this function. This can be any function that accepts two vectors as input and returns a single number. 
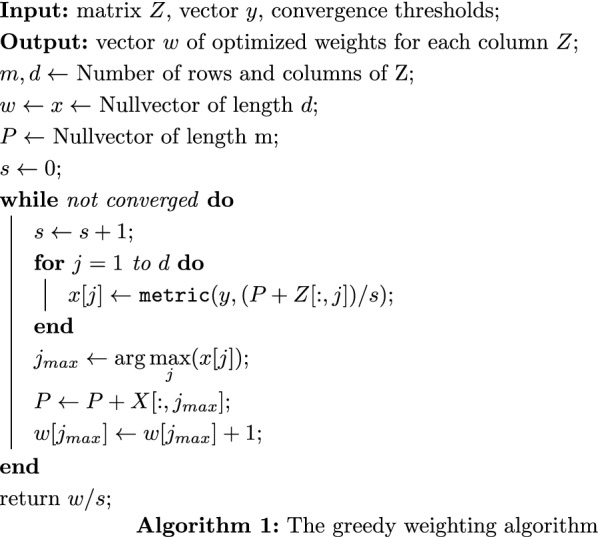


The algorithm works as follows: the vector *P* containing the greedy solution of the unnormalized weighted sum in each step is initialized with zero elements. All column weights and the total number of weights are also initialized to zero. In each iteration, first, the total number of weights is incremented. Then, all sums of *P* with a column of *Z* are normalized by the total number of weights and evaluated separately on the evaluation metric (i.e., AUROC, correlation). The column corresponding to the highest value is assigned one weight factor and added to *P*. This procedure is repeated *iter* times. Usually, setting *iter* to 100 is sufficient; higher values lead to increased precision but also increased computational time. The algorithm returns a vector of length *d*, with the number of columns of *Z*, containing weights for each column, summing to 1.

The fixed number of iterations *iter* can be derived as a convergence criterion. Common convergence criteria for iterative algorithms check whether the algorithm does not produce better results any more, or whether the solution found does not change any more. This is the case if the relative or absolute difference in the target function or the relative distance between the estimates of two subsequent steps falls below a threshold $\epsilon .$ Our algorithm estimates a parameter vector ${\mathbf {w}}$ iteratively with an estimate $\hat{\mathbf {w}}_{i}$ in step *i*. Using the relative difference of estimates with a percentage $\epsilon \in ]0,1[$ as convergence criterion, the algorithm is aborted after step *i*,  if $\Vert {\hat{\mathbf {w}}}_{i} - {\hat{\mathbf {w}}}_{i-1} \Vert < \epsilon \cdot \Vert \hat{\mathbf {w}}_{i-1}\Vert$. The components of $\mathbf {w}_i$ are positive integers summing to *i*. If the Manhattan distance ($L_1$-Norm) is used, the inequality thus evaluates to $1 <\epsilon \cdot i$, and with $\epsilon =0.01$ the algorithm is aborted after 100 iterations. If the euclidean distance ($L_2$-Norm) is used, convergence is achieved if $1<\epsilon \cdot \Vert \mathbf {w}_{i-1}\Vert _2.$ Because a problematic target function may differ dramatically for similar estimates of the parameter, the additional convergence of the target function is required in some cases. However, this is not the case in the examples presented.

In our application examples of this algorithm, we used seven different data sets, freely available at the UCI Machine Learning Repository [13]. All these data sets are real world examples that have been analyzed in previous publications. The *Mesotheliomas* data set predicts the presence of tumors based on socio-economic values and laboratory measurements [14]. In the *Lung Cancer* data set, three different types of lung cancer have to be predicted [15]. The *Diabetic Retinopathy* data set is again a binary classification task for the presence of this eye disease. In a similar way, the *Liver Disorder* data set predicts the presence of liver disease or not, but with very unbalanced classes [16]. We further feature two regression examples: first, the *Abalone* data set predicts the age of an abalone from physical measurements [17] and, second, the *Rand HIE* data set measures health care utilization costs from claims data [18]. The *Pima Indians Diabetes* data set is another well-known data set that predicts diabetes mellitus in a high risk population of Pima Indians in Arizona [19]. An overview of the data sets with respective number of observations, number of features, the prediction task (classification or regression), and the metrics we used to quantify the quality of the predictions is available in the Additional file 1. For classification tasks, we used accuracy (the percentage of making the correct prediction), the area under the receiver operator characteristic (AUROC), and the area under the precision recall curve (AUPR). For regression tasks, we chose the mean absolute error (MAE), i.e. the mean difference between observation and prediction, as the evaluation metric to be optimized.

For all classification tasks, we used three algorithms for ensembling: logistic regression, Random Forest, and a naive Bayes classifier [20]. The reason for selecting these algorithms is that they are based on completely different approaches and therefore make good candidates for ensembling as they may capture different aspects of the data sets: logistic regression is based on linear discrimination, Random Forest is based on decision trees and can apprehend complex interactions, and naive Bayes is a simple classifier using posterior probabilities based on Bayes’ rule. To analyze the behaviour of the greedy weighting in the presence of highly correlated predictions, we ensemble three Random Forest models for the Pima Indians data set that were calculated with different random seeds. This results in slightly different predictions but very high correlation (Spearman correlation coefficient $\rho >0.95$). For regression tasks, we used Random Forest regression, linear regression, and support vector regression (with radial kernel). Again, these algorithms provide different regression approaches, which make them ideal candidates for ensembling.

We compared the greedy weighting scheme with the brute force approach, i.e., all possible weighting combinations in steps of 0.01, and with a constrained linear model weighting. In this linear model with error term $\eta$,

$$\begin{aligned} Y = \beta _1 Z_1 + \beta _2 Z_2 + \cdots + \beta _j Z_j + \eta , \end{aligned}$$

the constraints $\sum _j \beta _j = 1$ and $\beta _j >0$ for the parameter estimates $\beta$ have to be satisfied to obtain valid weights. If specified as an optimization problem, it can be solved by quadratic programming, [21] i.e.,

$$\begin{aligned} \min \sum _i \left( Y_i - \left( \beta _{1} Z_{i1} + \beta _{2} Z_{i2} + \cdots + \beta _{j} Z_{ij} \right) \right) ^2. \end{aligned}$$

This is similar to the method in [3] and has the advantage of full interpretability of the weights as percentages. In addition, we compare it to the genetic stacking algorithm described in [5]. We evaluated all individual learners in a fivefold cross-validation setting. The linear, genetic, and greedy weighting scheme to optimally combine these individual predictions was assessed in an inner fivefold cross-validation by blending the predictions of multiple learners. The reported value is the average of all (outer) folds.

**Fig. 1:**
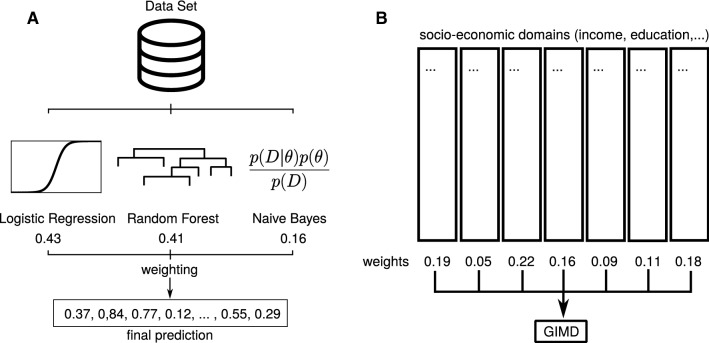
Schematic overview of both example cases for the greedy weighting algorithm. Numbers in the plots are just for illustration. **a** Logistic regression, Random Forest, and naive Bayes learners are combined to achieve a more accurate ensemble learner for classification. For regression tasks, Random Forest, linear regression, and support vector regression was used. **b** The GIMD is a weighted combination of different domains of deprivation

In a second application, we weighted the domains of the GIMD to be highly correlated with mortality, i.e., we maximized the Spearman correlation coefficient $\rho$ to the standardized mortality ratio (SMR). The SMR is the ratio of observed deaths in a municipal district to expected deaths in the same area. The GIMD (2010 version) is built upon seven different domains of deprivation (income, employment, education, municipal revenue, social capital, environment, and security) and covers all 412 districts of Germany (status 2010). The weighting of the seven domains is based on expert knowledge and follows the recommendations of Noble et al. [22]. More information on the GIMD can be found elsewhere [9, 10, 23]. We again compared this weighting with the constrained linear and brute force approaches. Figure 1 presents a graphical overview of both test cases.

A python implementation of the proposed algorithm, including code to reproduce the examples presented, is available online [24].

### Results

**Fig. 2:**
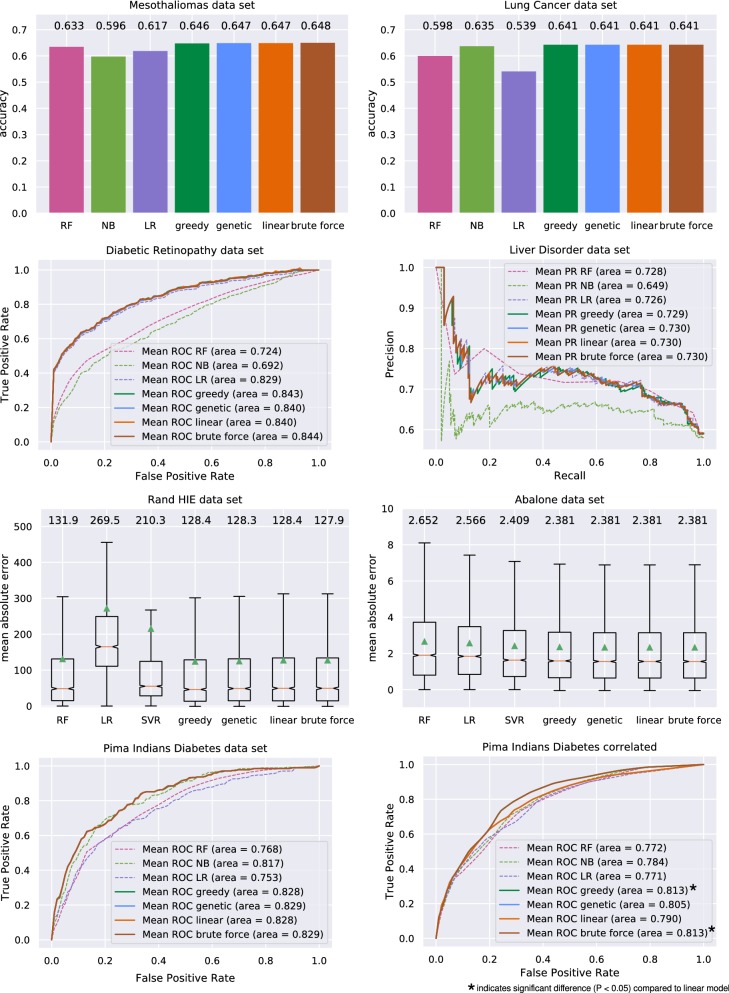
Results of the different weighting approaches for all data sets. Classification task include Random Forest (RF), naive Bayes (NB), and logistic regression (LR). Regression tasks are based on Random Forest, linear regression (LinR), and support vector regression (SVR). Stacking is based on the greedy, genetic, linear, and brute force methods

Figure 2 presents the results for all data sets. Generally, all stacked ensembling approaches outperform individual learners. Sometimes, the gain is only marginal, for example, the AUPR increase for the liver data set is only 0.002. The brute force approach usually provides the best weighting scheme, but the difference from the greedy, genetic, and linear weighting does not justify the huge computational effort required. For the lung cancer, diabetic retinopathy, and Pima Indians (correlated setting only) data sets, the brute force solution provides no advantage over the other stacking methods. The linear weighting is superior for the diabetic retinopathy and the Rand HIE data sets, but the greedy weighting produces higher or equal scores in all other cases. In the setting of correlated predictions for the Pima Indians data set, the advantage of the greedy weighting is very apparent with an AUROC of 0.813 compared with 0.790 for the linear weighting. Genetic algorithm weighting is very similar to linear and greedy in most cases. Because of the high number of possible combinations, the brute force ensemble takes 12 minutes to compute.

In Table 1, we compare the greedy weighting to the linear and genetic weighting. All methods are very fast ($<1$ second), but the results are quite different: the correlation with SMR is 0.615 for greedy weighting, 0.614 for genetic, and only 0.449 with linear. The baseline correlation, based on expert knowledge, is 0.578. The brute force approach takes 23 hours in this case.

**Table 1:** Comparison of different weighting approaches for correlation of GIMD domains with SMR

	SMR correlation	Computation time
Expert	0.578	NA
Brute force	0.616	23 h^a^
Greedy	0.615	$<1$ s
Genetic	0.614	$<1$ s
QP	0.449	$<1$ s

^a^This computation was performed on a high-performance computer

### Discussion

This paper demonstrates that a greedy approach provides a viable alternative for weighting different domains to a specific outcome. In the first case, we optimized the predictions of three different statistical learning algorithms to a combined prediction on several biomedical data sets. Here, the global optimum solution was often slightly better than the greedy approach, but at a cost: the brute force approach had to evaluate all $$\left( {\begin{array}{c}102\\ 2\end{array}}\right) =5,151$$ possible combinations. For a fair comparison, we restricted the analysis to candidate sets of values of length 3 (as we combine 3 learners) that sum to 1. Finding these sets is itself of exponential complexity as it is a variation of the subset sum problem [8]. Accordingly, the computation time of 12 minutes is not an accurate comparison measure because we omitted Random Forest hyperparameter tuning for each candidate set, as this would take unbearably long even on a HPC system. Although the linear ensemble is a fast and viable alternative, the greedy approach is superior in a setting with highly correlated predictions. Still, even in this setting, both the linear and genetic ensemble produces scores superior to the best single model.

In the second case, we optimized the domains of the GIMD to be highly correlated with SMR. Here, we obtained the actual domain weights to interpret them for the importance of regional mortality in future use. Interestingly, the linear approach here cannot optimize the weights as well as the greedy algorithm. Probably, the optimization is stuck in a local minimum here. Even the weighting scheme based on expert knowledge was more highly correlated with SMR. On this data set with 7 domains, the disadvantage of the brute force ensembling is very apparent: a brute force approach had to evaluate $$\left( {\begin{array}{c}106\\ 6\end{array}}\right) =1,705,904,746$$ possible combinations which took 23 h, parallelized on a HPC cluster.

## Limitations

While we tried to cover a wide range of data sets and scenarios in the biomedical field, results can be very different when applying the algorithm to other tasks or learners, or when other evaluations metrics are used.

## Supplementary information

**Additional file 1.** Derivation of convergence weights and description of data sets.

## Data Availability

The data sets in this study are freely available at the UCI machine learning repository (https://archive.ics.uci.edu/ml).

## Abbreviations

GIMD: German index of multiple deprivation
AUROC: Area under the receiver operator curve
AUPR: Area under the precision recall curve
MAE: Mean absolute error
SMR: Standardized mortality ratio
HPC: High performance computing
RF: Random Forest
NB: Naive Bayes
LR: Logistic regression
LinR: Linear regression
SVR: Support vector regression
TSP: Traveling salesman problem
