# Supplementary material for: A greedy stacking algorithm for model ensembling and domain weighting
Source: BMC Res Notes. 2020 Feb 12;13:70. doi: 10.1186/s13104-020-4931-7 (PMC7017540; doi:10.1186/s13104-020-4931-7)
Supplement: Supplementary file 1 — Additional file 1. Derivation of convergence weights and description of data sets. [file 13104_2020_4931_MOESM1_ESM.pdf]

Additional Files
Derivation convergence normalized weights
The weight vectors $\mathbf{w_{i-1}} = (w_{i-1}^1, \ldots, w_{i-1}^N)^T$ and $\mathbf{w_i} = (w_i^1, \ldots, w_i^N)^T$ in step $i-1$ and i are normalized by $i-1$ and i respectively. Therefore, the common denominator of each pair of vector components is $i(i-1)$. Let j_{max} be the index of the component that is incremented in step i. Then, $w_{i-1}^j = w_i^j$ for all $j \neq j_{\text{max}}$ and $w_i^{j_{\text{max}}} = w_{i-1}^{j_{\text{max}}} + 1$ and thus

$$\begin{aligned}
&\|\frac{1}{i}\mathbf{w_i} - \frac{1}{i-1}\mathbf{w_{i-1}}\|_1\\
=&\frac{1}{i(i-1)}\sum_{j=1}^{N}|(i-1)w_i^j - iw_{i-1}^j|\\
=&\frac{1}{i(i-1)}\sum_{j=1}^{N}|iw_i^j - iw_{i-1}^j - w_i^j|\\
=&\frac{1}{i(i-1)}(\sum_{j\neq j_{\text{max}}}|w_i^j| + |iw_i^{j_{\text{max}}} - iw_{i-1}^{j_{\text{max}}} - w_i^{j_{\text{max}}}|)\\
=&\frac{1}{i(i-1)}(i - w_{j_{\text{max}}} + |i - w_i^{j_{\text{max}}}|)\\
=&\frac{2(i-w_{j_{\text{max}}})}{i(i-1)}\\
=&2\frac{(1-\frac{w_{j_{\text{max}}}}{i})}{(i-1)}
\end{aligned}$$

Therefore, convergence is achieved if

$$\frac{1-\frac{w_{j_{\text{max}}}}{i}}{i-1} < \frac{\epsilon}{2}$$

extrema:
If $w_{j_{\text{max}}} = i$, this is always true.
If $w_{j_{\text{max}}} = 1$

$$\frac{1-\frac{1}{i}}{i-1} = \frac{i-1}{i(i-1)} = \frac{1}{i} < \frac{\epsilon}{2}$$

Data set	N	#features	task	metric
Mesotheliomas	324	5*	classification (binary)	accuracy
Lung cancer	32	56	classification (multiclass)	accuracy
Diabetic retinopathy	1,151	20	classification (binary)	AUROC
Liver disorder	345	7	classification (binary)	AUPR
Abalone	4,177	8	regression	MAE
Rand HIE	2,703	14	regression	MAE
Pima Indians diabetes	768	8	classification (binary)	AUROC

*the original data set has 34 features, but we only used the first five because of perfect discrimination otherwise.

Table S1 Description of data sets used, the prediction task, and the evaluation metric.
